# Anemia Diagnostic System Based on Impedance Measurement of Red Blood Cells

**DOI:** 10.3390/s21238043

**Published:** 2021-12-01

**Authors:** Hyuntae Cho, Seung-Ro Lee, Yunju Baek

**Affiliations:** 1 School of Digital Media Engineering, Tongmyong University, Busan 48520, Korea; marine@tu.ac.kr; 2 Division of Medical Product Safety, Daejeon Regional Office of Food and Drug Safety, Daejeon 35209, Korea; 3 School of Computer Science and Engineering, Pusan National University, Busan 46241, Korea

**Keywords:** anemia-measuring method, red blood cells, hemoglobin, hematocrit, in vitro system, blood diagnostics, test strip

## Abstract

Anemia is a condition in which red blood cells or the hemoglobin in the blood is lower than in healthy people. Red blood cells transport and supply oxygen needed to various organs in the human body. Anemia is caused by hypoxemia due to the lack of red blood cells and causes other serious health problems, such as heart problems, pregnancy complications, severe fatigue, or death. There are many causes of anemia, and it can be diagnosed by measuring hematocrit or hemoglobin levels in the blood. Even though there are various diagnostic devices on the market, these devices are inconvenient because their systems are bulky, heavy, expensive, or inaccurate. This study proposed a new anemia diagnostic system based on the impedance measurement of red blood cells. The proposed system consists of a test strip that collects a blood sample from the finger and a hemoglobin meter that measures the impedance of the blood and converts it into the concentration of hemoglobin. The proposed test strip that does not contain enzymes or reagents was designed in accordance with class 1 approval by the Food and Drug Administration (FDA). The hemoglobin meter was designed to include a hardware block, an algorithm block and a calibration block through empirical work. We also compared it to reference impedance to prove the accuracy of the hemoglobin meter. The experimental results with human blood indicated the superiority of the anemia diagnostic system. As a result, the overall standard deviation of impedance measurements was less than 1%, and the coefficient of variance of the proposed system was 1.7%, which was better than that of other commercial systems.

## 1. Introduction

Anemia is a condition where the hemoglobin concentration in the red blood cells is decreased. Red blood cells carry and supply oxygen to every organ in the body. As hemoglobin becomes insufficient, the oxygen-carrying ability of the blood decreases, resulting in the symptoms of a pale face and fatigue. Anemia exists in various forms as aplastic anemia, iron deficiency anemia [1], sickle cell anemia, thalassemia, vitamin deficiency anemia, and others, with the signs and symptoms varying depending upon the cause. The symptoms can include fatigue, cold hands and feet, weakness, headaches, dizziness, pale or yellow skin, and chest pain. Left untreated, anemia can cause more serious health problems, such as heart problems, pregnancy complications, severe fatigue, or death [2,3,4,5]. Anemia is usually diagnosed when the hemoglobin or hematocrit level is less than the normal threshold. Additional blood tests, endoscopy, and gynecological examinations can be used to check for the causes of diagnosed anemia. Anemia is a serious global public health problem that particularly affects young children and pregnant women. The world health organization (WHO) estimates that 42% of children younger than 5 years old and 40% of pregnant women worldwide are anemic [6]. Research and Markets Research Institute International estimated that the global hemoglobin testing market will reach $1.6 billion by 2027 [7].

An automatic hemoglobin meter is a type of in vitro diagnostic device used to manage diseases, such as polycythemia and anemia, and is used to test blood parameters, including hemoglobin concentration, leukocyte, and platelet counts. Automatic hemoglobin meters, which are mainly used when large hemoglobin measurement equipment is impractical, or in situations where rapid diagnosis is required, are used for onsite measurements using optical and electrochemical methods [8,9]. Nevertheless, the automatic hemoglobin meters to diagnose anemia, mostly used in developing countries, are still expensive and have some constraints, such as the expiration date of the enzymes or reagents.

In this study, we proposed an anemia diagnostic system based on the impedance measurement of red blood cells. We designed and manufactured a dedicated test strip to collect blood from the finger. The test strip does not contain the enzymes or reagents, so it complies with class 1 approval by the Food and Drug Administration (FDA). We also designed a dedicated system to measure the impedance of the blood in the cavity of the test strip and convert it into the concentration of hemoglobin. We evaluated the accuracy of the proposed anemia diagnostic system using ground truth reference impedance, then we evaluated its performance by measuring the blood of a human test group and comparing the values obtained to those of other meters. The proposed system had an impedance measurement error of less than 1% from the reference impedances and a coefficient of variance of 1.7%.

The rest of this paper is organized as follows. Section 2 introduces related works and the background of the proposed system. Section 3 describes how anemia is measured and how each function of the proposed system is implemented with detailed algorithms. Section 4 provides the experimental results of the proposed system. Finally, the conclusion of this paper is presented in Section 5 with recommendations for future studies.

## 2. Related Work

**Figure 1:**
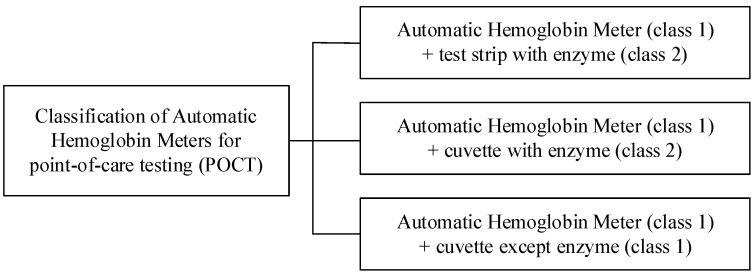
Classification of automatic hemoglobin meters for POCT.

The WHO describes the criteria to diagnose anemia from hemoglobin concentrations in the blood as shown in Table 1. In male adults, a hemoglobin concentration of less than 13 g/dL is considered anemia, while in females, anemia is considered when the hemoglobin concentration is less than 12 g/dL. Infants aged 6 months to 6 years and pregnant women are considered anemic if the hemoglobin concentration is less than 11 g/dL. To measure the hemoglobin concentration, automatic hemoglobin meters are widely used for point-of-care testing (POCT). Automatic hemoglobin meters for onsite testing are classified into three types as shown in Figure 1.

The automatic hemoglobin measuring system consists of a meter and a disposable microcuvette or test strip. Meters are controlled by the FDA with class 1 approval when they do not have enzymes or reagents. However, microcuvettes or test strips containing enzymes or reagents are managed by the FDA with class 2 approval, but the cuvette, which does not contain enzymes or reagents, falls under class 1 approval, as shown in Figure 1.

The automatic hemoglobin measurement systems using spectrophotometry consist of a portable measuring instrument (photometer) and a disposable plastic microcuvette. Currently, there are two types of automatic hemoglobin measurement systems, one using a microcuvette containing a reagent and another one measuring the amount of hemoglobin by measuring the absorbance of whole blood without using reagents. The HemoCue [10,11] and CompoLab TS [12] are representative examples. However, they are inconvenient because their systems are bulky, heavy, and expensive.

The electrochemical method converts the electrons generated during the reaction between ferrous (Fe^2+^) in hemoglobin and the electron transport medium in hemoglobin leaked after breaking the cell membrane of red blood cells in the blood into an electrical signal through an electrode. The generated current is converted to hemoglobin using an algorithm stored in the measuring instrument [13,14]. These instruments are smaller and cheaper than spectrophotometry systems. However, they are inaccurate and still require reagents to measure hemoglobin in the blood.

Some studies reported instruments that used up to five wavelengths ranging from 600 nm to 1400 nm for measuring hemoglobin concentrations and oxygen saturation. These multispectral measurement systems used the emission of laser light source for non-invasive measurement. In these investigations, the assembled sensor was fully integrated into a wearable finger clip [15,16]. In 2015, a low-cost point-of-care anemia detection device was proposed [17]. In a recent study, the hematocrit concentration was measured by blood impedance [18], and hemoglobin concentration measurements based on artificial intelligence were also reported [19].

## 3. Anemia Diagnostic System Based on Impedance Measurement

### 3.1. Strip Design

**Figure 2:**
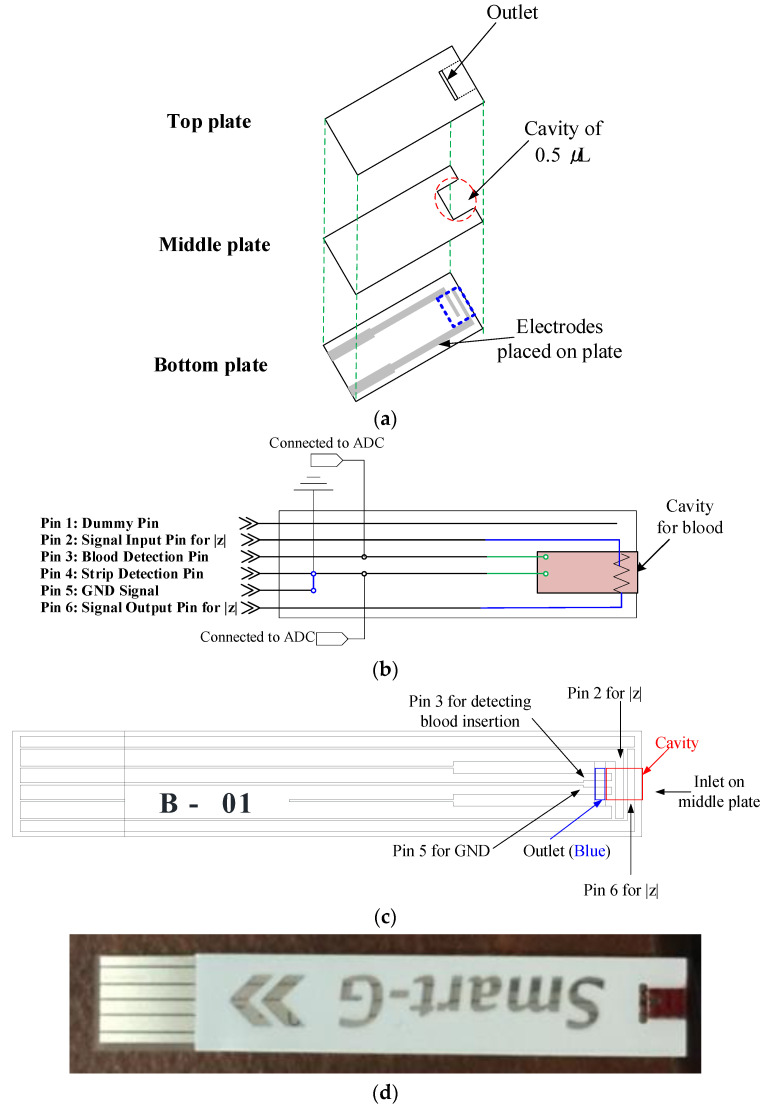
The designed strips: (**a**) structure of the strip, (**b**) electrodes of the test strip, (**c**) interface of the test strip, and (**d**) appearance of the test strip.

Figure 2a illustrates the test strip used for diagnosis, which consisted of three layers: the top, middle, and bottom plates. The top plate has an outlet that helps the strip absorb the blood quickly and easily, and the middle plate contains a 0.5 μL cavity. The bottom plate is composed of six electrodes to detect the insertion of the strip and blood, and blood impedance is measured as shown in Figure 2b. The system does not contain enzymes or reagents. We designed two strips, a test strip and a reference strip. The composition of the test strip is shown in Figure 2b, and it consists of six electrodes. Electrode 5 is connected to the GND of the anemia diagnostic meter, and electrode 4 is connected to electrode 5 for monitoring whether the test strip is inserted into the meter. Electrode 3 is used to check whether blood has flowed into the test strip, and electrodes 2 and 6 are used to measure the impedance of the blood. Electrode 1 is reserved for meter calibration. Figure 2c shows the structure of the strip in more detail. Electrode 3 is connected to 1.5 V and the analog-to-digital converter (ADC), and the other electrode is connected to the GND. When the blood enters the end of the cavity, the ADC value drops to a value close to that of the GND. The two electrodes for impedance measurement are placed between the blood as shown in Figure 2c.

The electrode material affects the performance of the entire system. Any change in the resistance and impedance of the material will make impedance measurements less consistent. When carbon is used as an electrode, it is difficult to uniformly distribute the material. That is, the uniformity decreases. For this reason, when measuring the impedance, part-to-part repeatability between strips is not guaranteed. An alternative is to use a material with a consistent impedance value. The electrodes in our system are based on Cu-Ni to provide strip-to-strip consistency.

**Figure 3:**
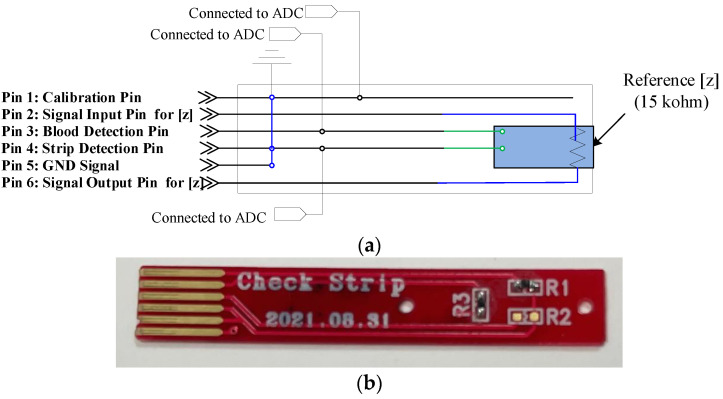
The manufactured test strip: (**a**) electrode structure and (**b**) appearance.

We designed a reference strip for the calibration of the measurement system. The basic operation of the reference strip is similar to that of the test strip, except for the dummy pin connected to the GND, which is used for meter calibration, as shown in Figure 3. If the reference strip is inserted into the anemia diagnostic meter, the meter will check the reference impedance (i.e., 15 kilo-ohms (kΩ)) mounted on the reference strip instead of the blood. This reference impedance of 15 kΩ is read by the meter initially, then the system derives a calibration factor from the difference between the real reference value and the measured value. This calibration factor is used for every measurement.

### 3.2. System Design of the Hemoglobin Meter

**Figure 4:**
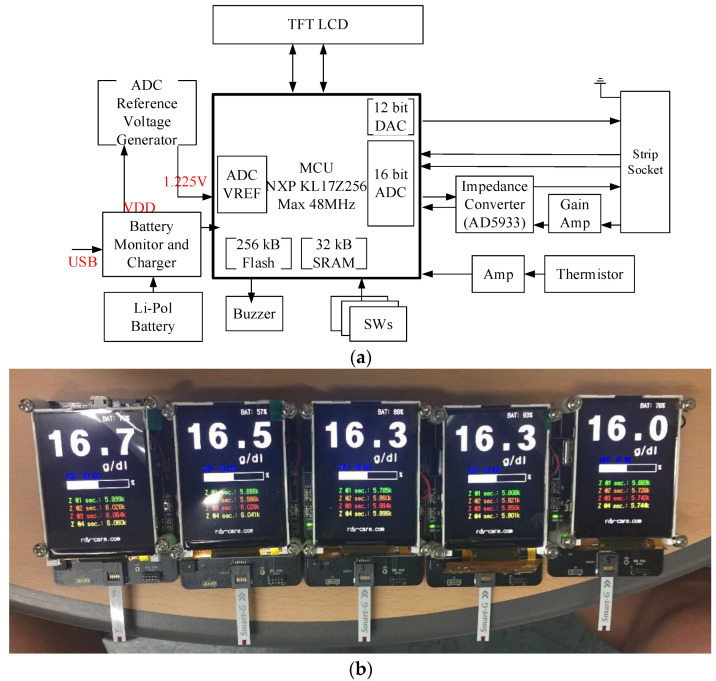
Hemoglobin meter: (**a**) block diagram and (**b**) prototypes (50 × 75 mm).

Figure 4 shows the overall block diagram of a hemoglobin meter based on impedance measurement. The system is powered by a battery that generates a low discharge current. Due to this constraint, NXP’s MKL17Z [20] microcontroller, a low-power MCU, controls the entire operation of the anemia diagnostic system. It can operate at a maximum speed of 48 MHz. However, power consumption is minimized by operating it at a low power mode of 4 MHz. It includes a built-in RTC to record the measured time and a 16-bit ADC and 12-bit digital-to-analog converter (DAC) required for blood detection and hemoglobin measurement.

Since the voltage generated by the internal battery can change over time, it can easily cause an error when used as a reference voltage for the anemia diagnostic system. The accuracy of the ADC and DAC is determined based on the voltage, and the result is linked to the accuracy of the whole system. To address this, we used a low-power shunt voltage reference [21] with an error of 0.1%, and the reference voltage used in the DAC and ADC was 1.225 V. To reduce the destruction of red blood cells due to high voltage, a low voltage was generated through the DAC and used to recognize the inflow of blood.

The hemoglobin meter should obtain the following information from the test strip: whether the test strip is inserted, whether the blood sample is introduced, and the impedance value of the blood sample for calculating the hemoglobin concentration. First, the meter can easily recognize whether the test strip was inserted and whether the blood sample was inflowed by reading the ADC values connected to the ADC pin.

**Figure 5:**
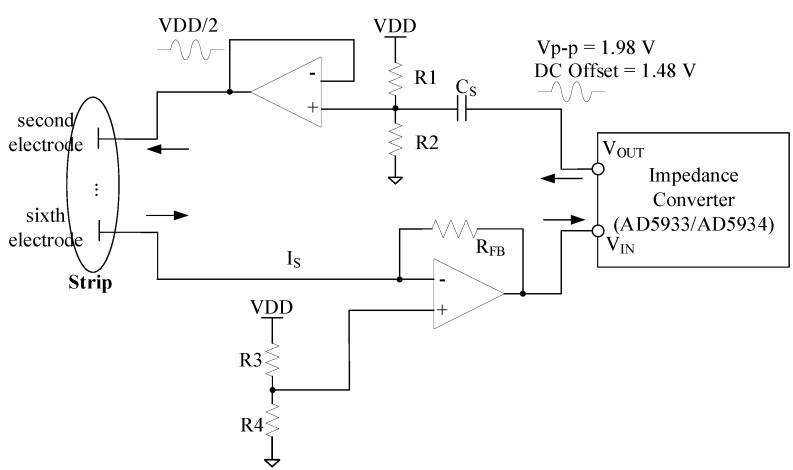
Impedance measurement circuit.

Next, to calculate the hemoglobin concentration, the impedance of the blood sample is measured first and converted to hemoglobin concentration. To calculate the impedance, this study used an analog device AD5933/34 impedance converter [22,23,24,25], which could be controlled through the I^2^C interface of the KL17Z MCU. Figure 5 shows a circuit for measuring an impedance greater than 1 kΩ. An OP-AMP was added to the transmitter (V_OUT_) to minimize interference, and a re-bias circuit was included to adjust the center of the signals. Also, a circuit for low leakage and low-noise current–voltage conversion was added to the receiver (V_IN_) stage. Adding the circuit allowed the AD5933/34 internal current-to-voltage converter (I-V converter) to act as a voltage follower.

In the circuit shown in Figure 5, R_FB_ is a parameter to amplify the output signal at the receiver stage and is an important factor determining the system performance. Since the reference of the internal ADC is equal to VDD, the value amplified by the trans-impedance amplifier (TIA) of the receiver should not exceed VDD. Otherwise, this value is saturated, and it is difficult to obtain a meaningful value above it. Therefore, it is necessary to select an appropriate R_FB_ value in consideration of this. This R_FB_ may be determined by the following equation:

(1) $$R_{\mathrm{FB}}=\frac{\left[\frac{\mathrm{VDD}}{2}-0.2\right]\times Z_{\mathrm{MIN}}}{\left[V_{\mathrm{PK}}+\frac{\mathrm{VDD}}{2}-V_{\mathrm{DCOFFSET}}\right]}\times \frac{1}{\mathrm{GAIN}}$$

where V_PK_ is the peak-to-peak voltage of the output signal, and one of the four ranges described in the datasheet [22] can be selected. In this study, we used 1.98 V Z_MIN_ as the minimum impedance value in the range of impedance to be measured, and the GAIN, which is the gain value of the amplifier in the AD5933/34 chip, was set to 1. VDD is the operating voltage value of the system, and V_DCOFFSET_ was designed to be the middle value of the operating voltage (VDD/2) as 1.5 V by re-biasing V_OUT_ with the DC offset voltage. As a result, a feedback resistance of 650 Ω was determined to measure impedances of 1 kΩ or greater.

To correct the error of the impedance converter, it is necessary to correct the offset after measuring it with the correct impedance value at the beginning. For this, the value of the reference impedance required for correction should be determined:

(2) $$R_{CAL}=\left(Z_{MIN}{+Z}_{MAX}\right)\times \frac{1}{3}$$

Since the measured impedances are taken in the range of 1 kΩ–50 kΩ (the values taken from the experiment conducted using blood), the gain factor of the impedance converter is initially obtained through the reference impedance of 15 kΩ. This gain factor calculated in Equation (3) was used to measure and correct the actual impedance:

(3) $$\mathrm{Gain}\;\mathrm{Factor}\;=\frac{\left(\frac{1}{\mathrm{Reference}\;\mathrm{Impedance}}\right)}{\mathrm{Magnitude}}$$

where magnitude is Re2+Im2, *Re* is a real value obtained through the discrete Fourier transform of the AD5933/34 impedance converter, and Im is an imaginary value. The system corrected the actual impedance as in Equation (4) when measuring the actual impedance through this gain factor:

(4) $$\left|Z\right|=\frac{1}{\mathrm{Gain}\;\mathrm{Factor}\;\times \;\mathrm{Magnitude}}$$

### 3.3. Software Operation of the Hemoglobin Meter

**Figure 6:**
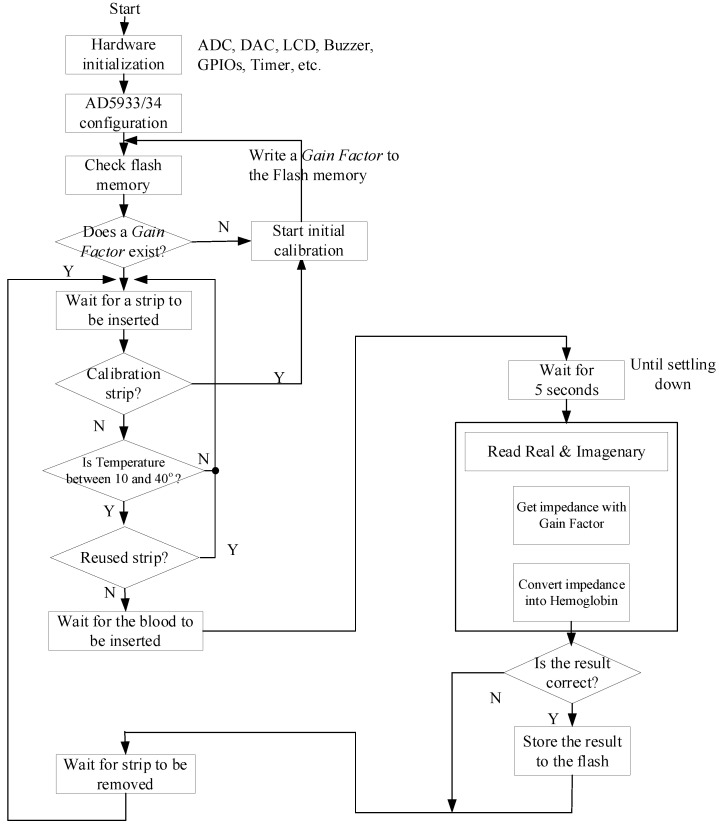
Flow chart of the system operation.

Figure 6 is a flowchart showing the software operation process. First, the peripheral hardware connected to the MCU is initialized and set to be operational, the necessary parameters of the AD5933/34 impedance converter for impedance measurement are set using the I^2^C interface, then the system checks the internal flash memory to see if the gain factor is written in the memory. This gain factor is measured and recorded in the flash memory through the reference strip shown in Figure 3 in the initialization procedure. If the gain factor value exists in the flash memory, it is read and used for impedance measurement. Otherwise, the initial correction process occurs, and the gain factor is calculated as in Equation (3). After reading the gain factor value, the system waits for the test strip to be inserted. If the inserted test strip is a reference strip for calibration, it undergoes an initial calibration process and checks the room temperature. If the room temperature is out of the range (10–40 °C), which is also used by the HemoCue device, it gives a warning and only operates within that range. After that, the system checks whether the inserted test strip is a contaminated test strip or a reused test strip, both of which have different ranges of impedance compared to new test strips. Contaminated test strips are identified based on this impedance range. If it is the correct test strip, the system confirms the entry of the blood sample and proceeds with the hemoglobin measurement process. To test the inflow of a blood sample, the MCU sets the general purpose input and output (GPIO) pin to ON. This pin is connected to the ADC channel. When no blood flows, the ADC has a high GPIO signal. When blood flows, it is shorted to the GND and has a low resistance, becoming easy to recognize. Once the blood is detected, this signal must be turned OFF to prevent effects from the voltage. For example, if DC voltage is continuously applied, heat may be generated and the blood may be dried more quickly. To prevent these effects, the impedance is measured using the AC voltage after cutting off the DC voltage.

After recognizing the blood sample and waiting 5 s in the idle state, the real part and the imaginary part are obtained through the AD5933/34, then the impedance value is obtained using Equation (4). At this time, to measure the impedance, a single frequency of 30 kHz is used without a frequency sweep [26,27,28]. The performance according to excitation frequency was well-described in the paper [18]. In that paper, a lower frequency from 10 to 1250 kHz showed better performance. The obtained impedance output value is converted into the hematocrit concentration (*Hct*) through a regression method [29,30].

*Hct (%) = a × |z| + b* (5)

where *a* is the gradient, *|z|* is the measured impedance, and *b* is the intercept.

Finally, the hemoglobin concentration is calculated by dividing the hematocrit by conversion ratio as shown in Equation (6) [31,32].

*Hb* (g/dL) = *Hct* (%)/conversion ratio (6)

## 4. Experimental Results

### 4.1. Hemoglobin vs. the Accuracy of the Measured Impedance

**Figure 7:**
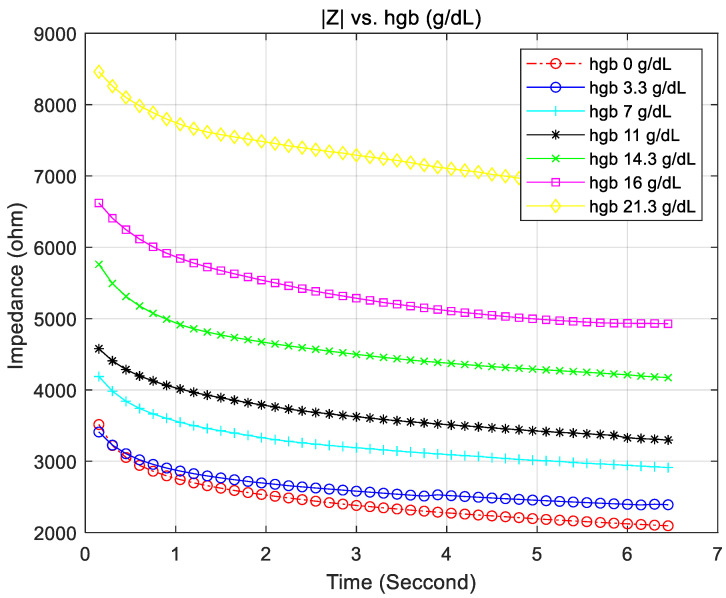
Impedance curve according to hemoglobin.

First, we observed whether the impedance of blood changed according to the hemoglobin concentration. For this experiment, the impedance according to the hemoglobin concentration was measured using the same test strip with a capacity of 0.5 μL to which no enzyme was adsorbed, as it may have acted as an interfering factor. The blood required for the experiment was prepared as follows. Venous blood was centrifuged, and the red blood cells were mixed with 100% plasma at the appropriate concentration. In this way, the hematocrit was obtained, and the hemoglobin concentration was calculated by dividing the hematocrit by three [31,32]. The blood samples used had hemoglobin concentrations of 0, 3.3, 7, 11, 14.3, 16, and 21.3 g/dL and were cross-validated with a reference instrument. Figure 7 shows that the impedance varied with the hemoglobin concentration and that the change curve became insensitive over time. In addition, we confirmed that the range of the measured impedance was within 2–9 kΩ. Based on this graph, when a blood sample was introduced and the signal was stabilized after 5 s, impedance was measured.

Before evaluating the performance of the whole system, we verified the accuracy of the proposed system to measure the impedance. Table 2 shows the impedance measurement accuracy of the blood glucose measurement device proposed in this paper. To evaluate the performance, we used six reference impedances, where the reference impedance was a cross-verified value through a high-performance instrument, and performed 200 repeated measurements for each impedance. As a result of the performance evaluation, the overall standard deviation was less than 1%, and the measurement range included all the ranges shown in Figure 7.

### 4.2. Performance Evaluation Using Human Blood

**Figure 8:**
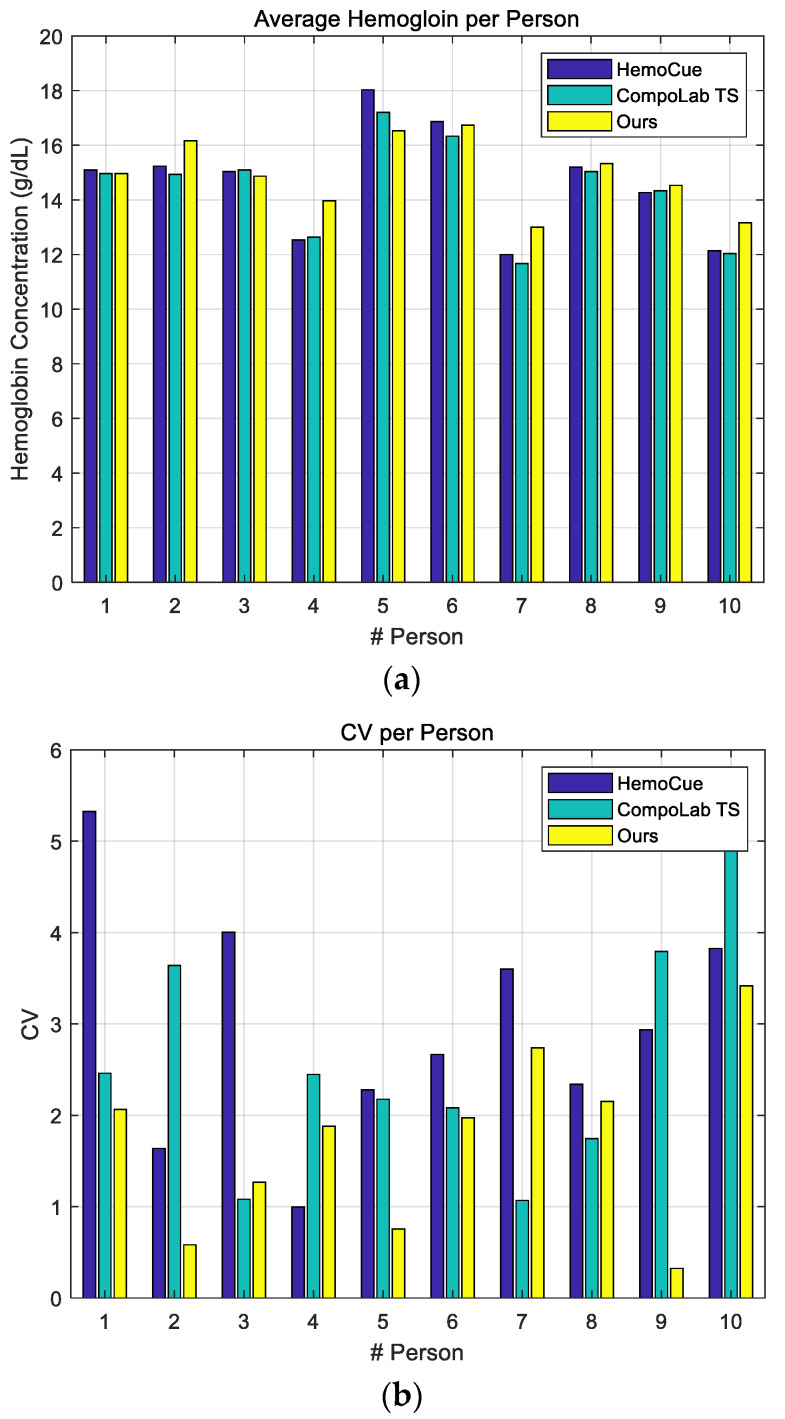
Experimental results using human blood: (**a**) average concentration and (**b**) CV.

The blood samples used in this evaluation were real human capillary blood and were used to compare and analyze the performance of the proposed system versus the HemoCue [10] and CompoLab TS [12] products. We conducted experiments on 10 people and conducted three tests for each person. Figure 8 shows the results, where Figure 8a shows the average concentration for each person measured by each instrument and Figure 8b shows the coefficient of variation (CV) of each instrument. As shown in Figure 8a, the average hemoglobin concentration per person for the instruments was almost equal. The average hemoglobin measurement of the first test subject was 15.1 g/dL for HemoCue, 14.97 g/dL for CompoLab TS, and 14.97 g/dL for the proposed system. For the fourth subject (with the largest error between systems), the measurements were 12.53 g/dL for HemoCue, 12.63 g/dL for CompoLab TS, and 13.97 g/dL for the proposed system. The difference between the proposed system and HemoCue was 1.44 g/dL. In actual in vitro diagnostic medical devices, the performance of the measuring device is determined through the CV rather than through the average measured value. The CV is calculated as *CV* = standard deviation/mean × 100. As shown in Figure 8b, the CV of the proposed system was lower than that of the existing systems. When measured three times from 10 people, the average CV of HemoCue, CompoLab TS, and the proposed system was 2.96%, 2.61%, and 1.7%, respectively. Also, the lowest CV recorded for HemoCue was 0.99%, CompoLab TS was 1.07%, and the proposed system was 0.32%. A lower CV means higher accuracy. These results indicated that the proposed method could estimate the concentration of hemoglobin well even if the impedance of blood was also affected by components other than red blood cells.

## 5. Conclusions

In this study, we designed a system to measure hemoglobin concentration. We took real human blood samples and measured the hemoglobin concentrations and impedances. To improve and calibrate the performance of the proposed system, we prepared blood samples with different hemoglobin concentrations, measured the impedance, and performed regression to convert the results to hemoglobin concentrations. The data obtained using venous blood had a small error between bloods, so higher accuracy was obtained compared to capillary blood. However, when collecting capillary blood, errors may increase due to various factors. We confirmed that the proposed system showed a lower CV value in the experiments and comparative analysis using capillary blood even in situations including such errors. The proposed system can also obtain class 1 approval from the FDA, as it does not need enzymes and reagents and can be easily used in developing countries due to its small size and remarkably reduced unit cost.

Future studies should include more experiments and evaluate hemoglobin concentration in other populations, including children and pregnant women. We will also perform evaluations based on variations in hemoglobin concentrations in capillary blood.

## Figures and Tables

**Table 1:** Criteria for low blood hemoglobin concentration.

Male Adult	<13 g/dL
Female Adult	<12 g/dL
Age 6~16 Years	<12 g/dL
Age 6 Months~6 Years	<11 g/dL
Pregnancy	<11 g/dL

**Table 2:** Impedance measurement accuracy.

**Reference |z|** **(Unit: Ω)**	680	0.98 k	1.96 k	20.18 k	32.8 k	50.58 k
**Mean of Measured |z| (Unit: Ω)**	695.3	0.998 k	1.99 k	20.34 k	32.8 k	49.91 k
**Std. Dev.** **(Unit: Ω)**	3.91	5.44	10.6	137.85	211.75	454.74
**Std. Dev.** **(Unit: %)**	0.58	0.55	0.54	0.68	0.65	0.9

## Data Availability

Not applicable.

## References

[1] Yap B.K., Soair M., Nur’Arifah S., Talik N.A., Lim W.F., Mei I.L. (2018). Potential Point-of-Care Microfluidic Devices to Diagnose Iron Deficiency Anemia. Sensors.

[2] Janz T.G., Johnson R.L., Rubenstein S.D. (2016). Anemia in the emergency department: Evaluation and treatment. Emerg. Med. Pract..

[3] GBD 2015 Disease and Injury Incidence and Prevalence Collaborators (2017). Global, regional, and national incidence, prevalence, and years lived with disability for 310 diseases and injuries, 1990–2015: A systematic analysis for the Global Burden of Disease Study 2015. Lancet.

[4] Tayles N. (1996). Anemia, genetic diseases, and malaria in prehistoric mainland Southeast Asia. Am. J. Phys. Anthropol..

[5] Mayo Foundation for Medical Education and Research Anemia. https://www.mayoclinic.org/diseases-conditions/anemia/symptoms-causes/syc-20351360.

[6] WHO Anemia. https://www.who.int/health-topics/anaemia#tab=tab_1.

[7] Research and Market The Hemoglobin Testing—Global Market Trajectory & Analytics. https://www.businesswire.com/news/home/20200903005654/en/Global-Hemoglobin-Testing-Industry-2020-to-2027---Market-Trajectory-Analytics---ResearchAndMarkets.com.

[8] Son Y., Park C., Min H., Lee I., Yoo S., Lee S., Kim B., Kim J. (2017). Safety and Testing Guideline for POCT Automatic Hemoglobin Meter.

[9] Srivastava T., Negandhi H., Neogi S.B., Sharma J., Saxena R. (2014). Methods for hemoglobin estimation: A review of “What Works”. J. Hemotol. Transf..

[10] HemoCue HemoCue Hb 301 System Manual. https://www.hemocue.com/en/solutions/hematology/hemocue-hb-301-system.

[11] Rippmann C.E., Nett P.C., Popovic D., Seifert B., Pasch T., Spahn D.R. (1997). HemoCue, an Accurate Bedside Method of Hemoglobin Measurement. J. Clin. Monit..

[12] Fresenuis Kabi CompoLab TS Manual. https://www.fresenius-kabi.com/in/products/compolab-ts.

[13] CeraGem Medisys (2014). Hb Plus Manual.

[14] BeneCheck BeneCheck Hb Manual; BeneCheck: 2021. https://palliance.se/wp-content/uploads/sites/6/2019/09/1150622205-Mission-plus-CE-Hb-Users-Manual-English-030816.pdf.

[15] Kraitl J., Timm U., Ewald H., Lewis E. Non-invasive sensor for an in vivo hemoglobin measurement. Proceedings of the IEEE Sensors.

[16] Timm U., McGrath D., Lewis E., Kraitl J., Ewald H. Sensor system for non-invasive optical hemoglobin determination. Proceedings of the IEEE Sensors.

[17] Punter-Villagrasa J., Cid J., Páez-Avilés C., Rodríguez-Villarreal I., Juanola-Feliu E., Colomer-Farrarons J., Miribel-Català P.L. (2015). An Instantaneous Low-Cost Point-of-Care Anemia Detection Device. Sensors.

[18] Chakraborty S., Das S., Das C., Chandra S., Sharma K.D., Karmakar A., Chattoapadhyay S. (2020). On-chip estimation of hematocrit level for diagnosing anemic conditions by Impedimetric techniques. Biomed. Microdev..

[19] Reis G., Tan X., Kraft L., Yilmaz M., Schoeb D.S., Miernik A. (2021). Safe Hb Concentration Measurement during Bladder Irrigation Using Artificial Intelligence. Sensors.

[20] NXP NXP KL17Z Datasheet. https://www.nxp.com.

[21] TI (2016). LM4041-N Precision Micropower Shunt Voltage Reference Datasheet. https://www.ti.com.

[22] Analog Devices AD5933 Impedance Converter Datasheet. https://www.analog.com/en/products/ad5933.html.

[23] Analog Devices Circuit Note CN-0217. https://www.analog.com/en/design-center/reference-designs/circuits-from-the-lab/cn0217.html.

[24] Cho H. (2020). Design and Implementation of a Blood-Glucose Meter to Reduce Hematocrit Interference. IEMEK J. Embed. Syst. Appl..

[25] Shimpi P.D., Yadav D.M. (2015). Bio-impedance Detection using AD5933 Impedance Converter Analyzer. Int. J. Sci. Res..

[26] Analog Device Evaluation Board User Guide: UG-364. https://www.analog.com/media/en/technical-documentation/user-guides/ug-364.pdf.

[27] Djermanova N.J., Kiss’ovski J.G., Vatchkov V.A. (2014). Portable Arduino-Based LCR–Meter. Annu. J. Electron..

[28] Chabowski K., Piasecki T., Dzierka A., Nitsch K. (2015). Simple wide frequency range impedance meter based on ad5933 integrated circuit. Metrol. Meas. Syst..

[29] Aalen O.O. (1989). A linear regression model for the analysis of life times. Stat. Med..

[30] Yao W., Longhai L. (2014). A new regression model: Modal linear regression. Scand. J. Stat..

[31] Carneiro I.A., Drakeley C.J., Owusu-Agyei S., Mmbando B., Chandramohan D. (2007). Haemoglobin and haematocrit: Is the threefold conversion valid for assessing anaemia in malaria-endemic settings?. Malar. J..

[32] Rudolf J., Douglass J., Baron J., Lewandrowski K. (2015). Evaluation of the i-STAT point-of-care capillary whole blood hematocrit and hemoglobin: Comparison to the Siemens RAPIDLab 1200, Sysmex XE5000, and manual spun hematocrit. Clin. Chim. Acta.

